# A Randomized Controlled Trial Comparing Low-FODMAP and Gluten-Free Diets on Gastrointestinal Symptoms, Nutritional Status, and Quality of Life in Irritable Bowel Syndrome

**DOI:** 10.5152/tjg.2026.26147

**Published:** 2026-06-25

**Authors:** Adeviye Eda Somtaş, Aslı Gizem Çapar

**Affiliations:** Department of Nutrition and Dietetics, Nuh Naci Yazgan University Faculty of Health Sciences, Kayseri, Türkiye

**Keywords:** Functional gastrointestinal disorders, gluten-free diet, irritable bowel syndrome, low-FODMAP diet, nutrition therapy, randomized controlled trial

## Abstract

**Background/Aims:**

: Irritable bowel syndrome (IBS) is a common chronic gastrointestinal disorder that requires a symptom-based treatment approach, in which dietary and lifestyle modifications play a central role in reducing symptoms and improving quality of life. Therefore, dietary strategies should be individualized. This study compared the short-term effects of low-fermentable oligosaccharides, disaccharides, monosaccharides, and polyols (FODMAP) and gluten-free diets on symptom severity, health-related quality of life, dietary intake, and anthropometric measurements in individuals with IBS.

**Materials and Methods:**

In this 3-arm, parallel-group randomized controlled trial, 63 adults diagnosed with IBS according to the Rome IV criteria were enrolled. Participants were randomly assigned to a low-FODMAP diet (n = 21), a gluten-free diet (n = 21), or a control group (n = 21). The intervention period lasted 4 weeks. Assessments were conducted at baseline and after the intervention using the IBS Symptom Severity Score (IBS-SSS), IBS Quality of Life questionnaire (IBS-QOL), IBS Visual Analog Scale, 3-day dietary intake records, and anthropometric measurements.

**Results:**

Both intervention groups demonstrated reductions in IBS-SSS scores and improvements in IBS-QOL scores (*P* < .001). After adjustment for baseline values, a significant between-group difference was observed in quality of life (*P* < .001), whereas no significant difference was found in symptom severity (*P* > .05). Significant between-group differences were also observed in body weight, body mass index, waist circumference, waist-to-hip ratio, and carbohydrate intake.

**Conclusion:**

Low-FODMAP and gluten-free diets may improve quality of life in individuals with IBS in the short term. After adjustment for baseline values, a between-group difference was observed only in quality of life, whereas no difference was found in symptom severity. These findings suggest that both dietary interventions may represent effective nutritional strategies for the management of IBS.

**Main Points**

- Both low-FODMAP and gluten-free diets are effective in reducing gastrointestinal symptom severity and improving quality of life in individuals with irritable bowel syndrome.
- The low-FODMAP diet is associated with more comprehensive improvements in anthropometric measurements, including waist circumference and waist-to-hip ratio.
- Because of the risk of nutrient deficiencies and individual variability, dietary interventions should be monitored under the supervision of a qualified dietitian.

## Introduction

Irritable bowel syndrome (IBS) is a functional gastrointestinal disorder characterized by recurrent abdominal pain associated with altered bowel habits.^1^ The global prevalence of IBS ranges from 3.8% to 15%, whereas prevalence estimates in Türkiye vary from 6.2% to 19.1%, reflecting differences in diagnostic criteria and study populations.^2^^-^^4^ Although the pathophysiology of IBS has not been fully elucidated, it is widely recognized as a multifactorial disorder involving genetic susceptibility, environmental influences, dietary patterns, alterations in gut microbiota, low-grade inflammation, and psychosocial factors.^5^^,^^6^ Symptom heterogeneity and variability in diagnostic criteria further complicate disease management, and current therapeutic strategies primarily focus on symptom control and improvement of health-related quality of life rather than on curative treatment.^7^Dietary factors are among the most frequently reported triggers of IBS symptoms, positioning nutrition a cornerstone of disease management.^8^ Consequently, national and international guidelines recommend individualized, balanced dietary interventions as first-line therapy.^9^^,^^10^ Among these approaches, the low-FODMAP diet has the strongest evidence base and has been shown to reduce abdominal pain, bloating, and flatulence by restricting fermentable short-chain carbohydrates.^11^ Randomized controlled trials have reported symptom improvement in up to 80% of patients within 6 weeks.^12^ Gluten-free diets may benefit a subset of patients with IBS and suspected non-celiac gluten sensitivity; however, in the absence of celiac disease, symptom improvement is often attributed to fructan restriction and placebo effects rather than to gluten elimination itself.^13^ Elimination-based dietary interventions should be implemented under the supervision of a dietitian to ensure nutritional adequacy and long-term sustainability. Individualized nutritional education has been shown to improve symptom awareness, self-management, and quality of life in patients with IBS.^14^ In the literature, the effectiveness of low-FODMAP and gluten-free diets in IBS has largely been investigated separately, and randomized controlled studies directly comparing these 2 approaches using disease-specific validated instruments remain limited. This study aimed to address this gap by evaluating the effects of low-FODMAP and gluten-free diets on IBS symptoms and quality of life within the same methodological framework. The findings may provide clinically relevant evidence to support evidence-based, personalized nutritional strategies for the management of IBS.

## Materials and Methods

### Study Design and Participants

This study was designed as a three3-arm, parallel-group, randomized controlled intervention trial and was conducted between June 2023 and April 2024 among individuals diagnosed with IBS who attending the Gastroenterology Outpatient Clinic of Kayseri City Hospital. Written informed consent was obtained from all participants beforeprior to enrollment. The study protocol was approved by the Scientific Research and Publication Ethics Committee of Nuh Naci Yazgan University (April 27, 2023; decision no: 2023/005-001) and conducted in accordance with the principles of Declaration of Helsinki. Institutional approval permission was obtained from Kayseri City Hospital (decision no: 72; June 7, 2023). Eligible participants were adults aged 18-65 years who had been diagnosed with IBS according to the Rome IV criteria by a gastroenterologist and had normal abdominal ultrasonography findings. Exclusion criteria included inflammatory bowel disease, celiac disease, lactose malabsorption, metabolic disorders (e.g., cardiovascular disease or diabetes mellitus), acute infection or fever, history of gastrointestinal surgery or malignancy, pregnancy or lactation, unintentional weight loss ≥5 kg during the previous month, probiotic use within the last month, and adherence to a gluten-free or low-FODMAP diet during the previous month.No generative artificial intelligence (AI) tools were used in the writing, analysis, interpretation, or preparation of this manuscript. This study was registered at ClinicalTrials.gov (Identifier: NCT06117072).

### Randomization, Blinding, and Sample Size

**Figure 1.**
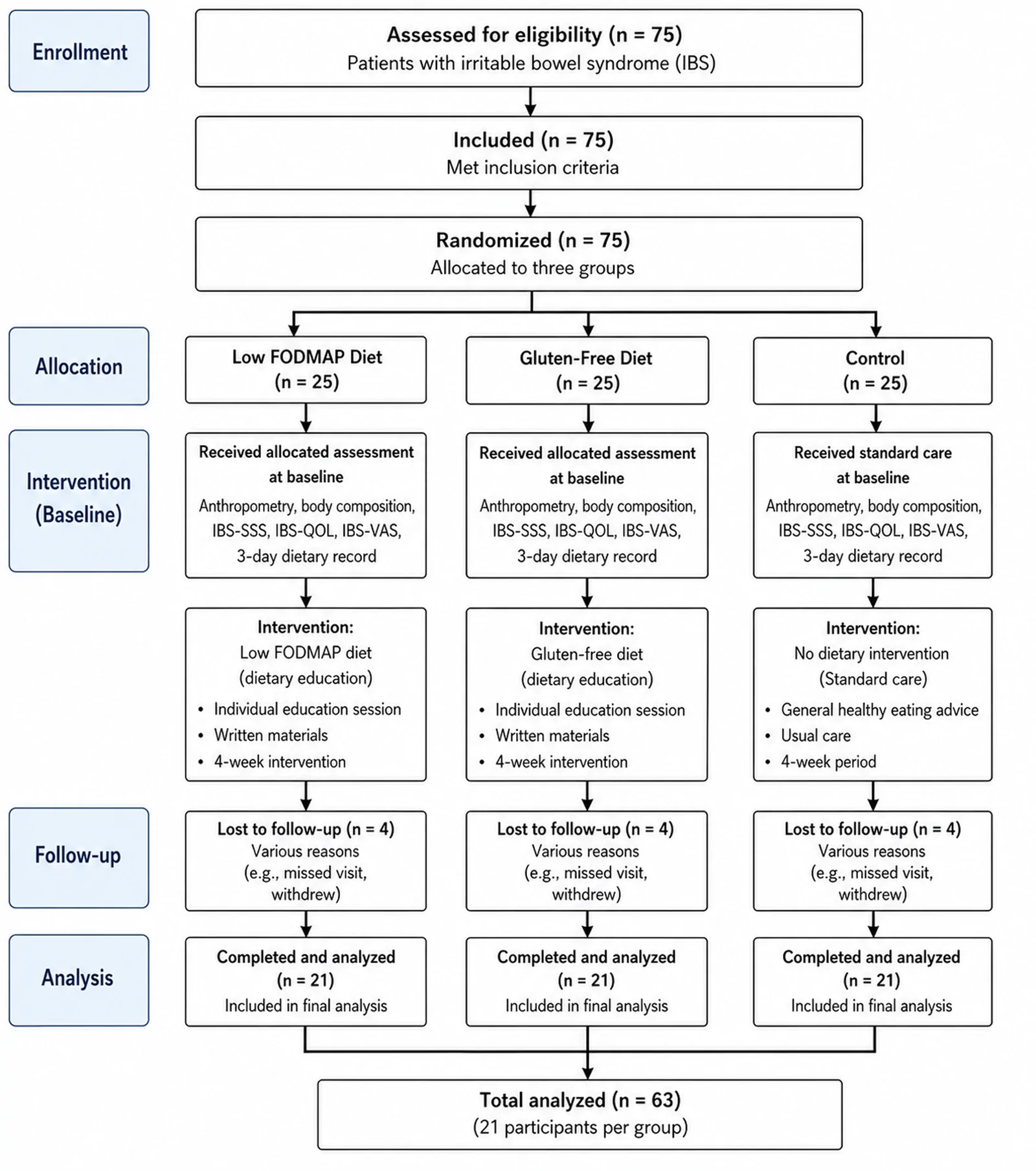
Flow diagram of participant recruitment, randomization, follow-up, and analysis. A total of 75 participants with irritable bowel syndrome were assessed for eligibility and met the inclusion criteria. All participants were randomized into 3 groups: low-FODMAP diet (n = 25), gluten-free diet (n = 25), and control group (n = 25). Baseline assessments included anthropometry, body composition, Irritable Bowel Syndrome Symptom Severity Score, Irritable Bowel Syndrome Quality of Life questionnaire, Irritable Bowel Syndrome Visual Analog Scale, and 3-day dietary records. During the follow-up period, 4 participants from each group were lost to follow-up for various reasons. Consequently, 21 participants in each group completed the study and were included in the final analysis. Follow-up assessments were conducted at week 4 using the same outcome measures.

Participants were randomly allocated in a 1 : 1 : 1 ratio to the control, low-FODMAP diet, or gluten-free diet groups using a computer-generated random number sequence (n = 21 per group). The randomization sequence was generated by an independent researcher, and allocation concealment was maintained until assignment. No dietary intervention was administered to the control group. Because of the nature of dietary interventions, blinding of participants and investigators was not feasible; however, standardized, validated outcome measures were used to minimize assessment bias. Based on Paduano et al,^15^ sample size calculations assumed an effect size of 0.886, 80% power, and a 2-sided *α* of 0.05, requiring at least 21 participants per group. A total of 75 patients with IBS were assessed for eligibility, and all met the inclusion criteria and were subsequently enrolled in the study. Participants were randomly assigned using a computer-generated randomization sequence to the low-FODMAP diet (n = 25), gluten-free diet (n = 25), and control (n = 25) groups. At baseline, all participants commenced their assigned intervention protocols (dietary education or standard care). During the follow-up period, 12 participants were lost to follow-up for various reasons (e.g., inability to attend scheduled visits, unwillingness to continue the intervention, or voluntary withdrawal), with equal distribution across groups (n = 4 per group). Consequently, 63 participants completed the study, and 21 participants from each group were included in the final statistical analysis. The Consolidated Standards of Reporting Trials (CONSORT) flow diagram of participant progression through the study is presented in Figure 1.

### Data Collection and Outcome Measures

Data were collected by a trained research dietitian through face-to-face interviews at baseline (week 1) and after the intervention (week 4). Baseline assessments included sociodemographic characteristics, dietary habits, physical activity using the International Physical Activity Questionnaire - Short Form (IPAQ-SF), a 3-day retrospective dietary intake record, IBS Symptom Severity Score (IBS-SSS), IBS Quality of Life questionnaire (IBS-QOL), and IBS Visual Analog Scale (IBS-VAS). All assessments were repeated at follow-up using identical procedures.

### Anthropometric Measurements

Body weight and height were measured using a stadiometer (SECA 213, Finland). Body mass index (BMI), basal metabolic rate, and body composition were assessed using a bipolar bioelectrical impedance analyzer (ACCUNIQ Jawon BC-310, South Korea).

### Dietary Intake Assessment

Three-day dietary intake records (2 weekdays and 1 weekend day) were obtained via face-to-face interviews at baseline and follow-up. Portion sizes were estimated using the Food and Nutrition Photograph Catalogue.^16^ Energy and macro- and micronutrient intakes were analyzed using the Nutrition Information Systems Software Package (BEBIS version 8.1).

### Irritable Bowel Syndrome Symptom Severity Score

IBS symptom severity was assessed using the IBS-SSS.^17^ The scale yields a total score of 0-500, classifying symptoms as remission (0-74), mild (75-174), moderate (175-299), or severe (≥300). A reduction of ≥50 points was considered clinically meaningful.

### Irritable Bowel Syndrome Quality of Life Questionnaire

Health-related quality of life was evaluated using the IBS-QOL questionnaire,^18^ validated in Turkish by Uran et al.^19^ Total scores range from 34 to 170, with higher scores indicating better quality of life; an increase of ≥10 points was considered clinically meaningful.

### Irritable Bowel Syndrome Visual Analog Scale

IBS-related symptoms were assessed using an IBS-adapted Visual Analog Scale.^20^Multiple gastrointestinal and psychosocial symptoms were rated on a 100-mm visual analog line, with higher scores indicating greater symptom severity. Although the VAS is conventionally scored on a continuous 0-100 mm scale, scores in this study were converted into a 10-point ordinal scale (1 = very poor, 10 = very good) to facilitate clinical interpretation and improve practicality in analysis. Participants marked their symptom status on the line, and scores were subsequently categorized for analysis.

### Dietary Intervention

Participants followed either a low-FODMAP diet or a gluten-free diet for 4 weeks according to group allocation. Before the intervention, all participants received standardized IBS education and individualized dietary counseling lasting 45-60 minutes. Dietary adherence was supported through weekly telephone contacts and a face-to-face follow-up visit at week 4. The gluten-free diet was implemented according to current recommendations and involved avoiding wheat, barley, rye, bread, pasta, bulgur, baked goods, and other processed foods containing gluten while encouraging naturally gluten-free foods such as rice, corn, quinoa, buckwheat, meat, fish, eggs, fruits, and vegetables.^21^^,^^22^ The low-FODMAP diet involved restricting fermentable carbohydrates, including fructans, galacto-oligosaccharides, lactose, excess fructose, and polyols. Participants were encouraged to consume bananas, citrus fruits, strawberries, carrots, zucchini, eggplant, and lactose-free dairy products while avoiding apples, pears, onions, garlic, legumes, and wheat-based products.^22^^,^^23^ Dietary adherence was indirectly assessed using 3-day dietary records collected at baseline and week 4.

### Physical Activity Assessment

Physical activity levels were assessed at baseline using the Turkish-validated short form of the IPAQ-SF and expressed as metabolic equivalent of task (MET)-min/week.^24^

### Statistical Analysis

The normality of the data was assessed using histograms, Q–Q plots, and the Shapiro–Wilk test. Homogeneity of variances was evaluated using Levene’s test. The Pearson’s *χ*^2^ test was used for comparisons of categorical variables. For repeated paired measurements, the paired samples *t*-test and Wilcoxon signed-rank test were used for quantitative variables. For comparisons among more than 2 groups, 1-way analysis of variance and the Kruskal–Wallis test were used. Bonferroni and Tukey post hoc tests were applied for multiple comparisons. After controlling for baseline measurements, the effects on postintervention measurements were investigated using analysis of covariance (ANCOVA). Data were analyzed using R version 4.5.1 (www.r-project.org) (R Core Team, R Foundation for Statistical Computing; Vienna, Austria). A *P*-value < .05 was considered statistically significant.

## Results

### Sociodemographic Characteristics

Baseline sociodemographic characteristics, including sex, marital status, education, economic status, employment, smoking, alcohol consumption, and physical activity levels, did not differ significantly between groups (Table 1). Median age was comparable between groups (low FODMAP = 26 (23-36.5) years; gluten free = 26 (24-34.5) years). There was no significant difference in age between the groups (*P* = .838).

### Anthropometric Measurements

At baseline, anthropometric measurements were comparable between groups (*P* > .05). Within-group analyses showed significant reductions in body weight, BMI, waist circumference, and waist-to-hip ratio (WHR) in the low-FODMAP diet group (*P* = .006, *P* = .012, *P* < .001, and *P* = .025, respectively). In the gluten-free diet group, a significant reduction was observed only in body weight (*P* = .040) (Table 2). After adjustment for baseline values, ANCOVA analysis revealed statistically significant differences between groups in body weight (*F* = 5.188, *P* = .008, *η*^2^ = 0.150), BMI (*F* = 3.581, *P* = .034, *η*^2^ = 0.108), waist circumference (*F* = 4.502, *P* = .015, *η*^2^ = 0.132),and WHR (*F* = 3.775, *P* = .029, *η*^2^ = 0.113).

### IBS-Specific Quality of Life and Symptom Severity

**Figure 2.**
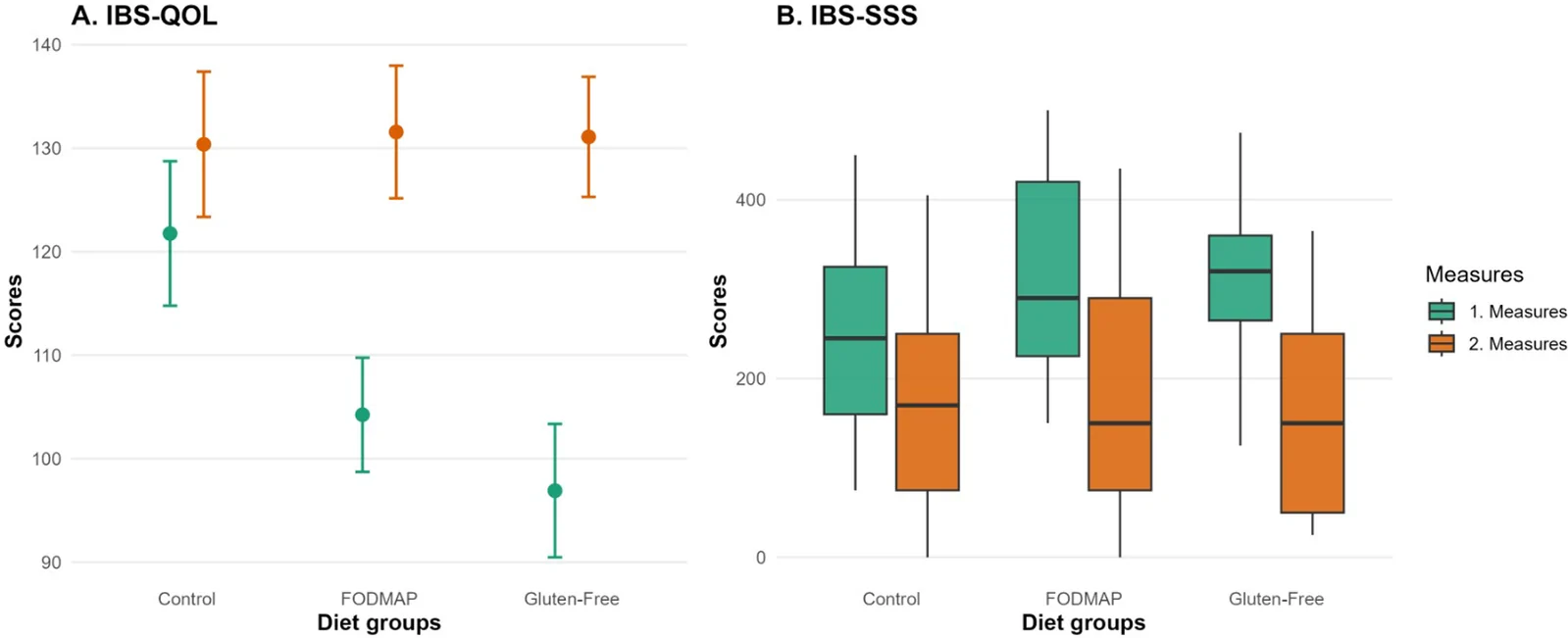
Changes in irritable bowel syndrome Quality of Life questionnaire (IBS-QOL) and irritable bowel syndrome Symptom Severity Score (IBS-SSS) scores before and after the intervention across study groups. (A) Comparison of IBS-QOL scores between baseline and pos-intervention across control, FODMAP diet, and gluten-free diet groups. (B) Distribution of IBS-SSS scores at baseline and postintervention across the 3 groups.

At baseline, IBS-QOL scores differed significantly between groups (*P* = .022), with lower scores in the gluten-free diet group than in the control group. At week 4, IBS-QOL scores increased significantly in all groups (all *P* < .001), with no difference between the FODMAP and gluten-free diet groups. After adjustment for baseline values, ANCOVA showed a significant between-group difference in IBS-QOL (F = 7.909, *P* < .001, *η*^2^ = 0.211). IBS-SSS scores decreased significantly in both the low-FODMAP and gluten-free diet groups (both *P* < .001) and improved over time across all groups (all *P* < .001). However, after baseline adjustment, no significant between-group difference was observed for IBS-SSS (F = 2.252, *P* = .114, η^2^ = 0.071) (Table 2, Figure 2).

### Dietary Energy, Macronutrient, and Fiber Intakes

At baseline, significant between-group differences were observed for energy (*P* = .014), protein (*P* = .034), and fat intake (*P* = .041). At week 4, only carbohydrate intake differed significantly between groups (*P* < .001), with higher intake in the control group. Within-group analyses demonstrated reduced carbohydrate intake in both the low-FODMAP (*P* = .017) and gluten-free diet groups (*P* < .001), increased protein intake in the low-FODMAP diet group (*P* = .003), and reduced energy intake in the gluten-free diet group (*P* = .027). After adjustment for baseline values using ANCOVA, a significant between-group difference remained for carbohydrate intake only (*F* = 5.166, *P* = .009, *η*^2^ = 0.149) (Table 3).

### IBS Symptom Severity (Visual Analog Scale)

At baseline, significant between-group differences were observed for abdominal pain (*P* = .008), constipation (*P* = .048), and bloating/gas (*P* = .008). At week 4, bloating/gas remained the only variable that differed significantly between groups (*P* = .033). Within-group analyses demonstrated increases in abdominal pain, constipation, and bloating/gas in both intervention groups (all *P* ≤ .008), accompanied by worsening psychological status (*P* ≤ .009). After adjustment for baseline values using ANCOVA, significant between-group differences were observed for abdominal pain (*P* = .021, *η*^2^ = 0.122), constipation (*P* = .002, *η*^2^ = 0.185), bloating/gas (*P* = .002, *η*^2^ = 0.192), and psychological status (*P* = .005, *η*^2^ = 0.166) (Table 4).

## Discussion

This randomized controlled intervention study evaluated the short-term effects of low-FODMAP and gluten-free diets in patients with IBS. Both interventions were associated with significant improvements in symptom severity and quality of life after 4 weeks. After adjustment for baseline values, a significant between-group difference was observed for quality of life, whereas symptom severity did not differ significantly between groups. These findings suggest both dietary approaches provide comparable effects on symptom control, although their effects on quality of life may differ. Consistent with these findings, a study conducted in the country demonstrated significant symptom improvement across all IBS subtypes following a low-FODMAP diet combined with probiotic supplementation; however, probiotics did not confer additional benefits regarding symptom response or dietary adherence, suggesting that the primary therapeutic effect may be largely attributable to dietary modification itself.^25^

Individuals with IBS have been reported to have lower quality of life than healthy individuals.^26^Therefore, dietary interventions have emerged as an important strategy for improving symptom control and quality of life.^27^ A study conducted in Türkiye demonstrated that a low-FODMAP diet was associated with significant improvements in IBS-QOL scores among women with IBS.^28^ Similarly, a randomized controlled trial involving 84 patients with IBS reported that a low-FODMAP diet significantly improved quality of life, particularly among patients with IBS-D.^29^ Furthermore, a comparative study evaluating low-FODMAP, gluten-free, and Mediterranean diets demonstrated significant postintervention improvements in quality of life across all dietary groups.^15^ Similarly, in the present study, IBS-QOL scores increased significantly over time in all groups. At baseline, a statistically significant between-group difference was observed, with lower scores in the gluten-free diet group than in the control group. Although no significant difference was detected between the FODMAP and gluten-free diet groups at week 4, the between-group difference in IBS-QOL remained statistically significant after adjustment for baseline values. These findings indicate that, despite overall improvements in quality of life across all groups, baseline differences between groups may have influenced the outcomes. The persistence of between-group differences after adjustment for baseline values suggests that the observed variation in quality of life cannot be attributed solely to the intervention effects and may also reflect the influence of methodological and individual-level factors.

Current evidence-based guidelines emphasize that the management of IBS should be individualized and symptom oriented. The American College of Gastroenterology guideline recommends a positive diagnostic approach based on the Rome IV criteria and identifies dietary modification, particularly a low-FODMAP diet, as an effective first-line strategy for short-term symptom management.^1^^,^^23^ Similarly, the British Society of Gastroenterology guideline supports structured dietary interventions within a multidisciplinary framework.^1^ More recent evidence from the Seoul Consensus on IBS further supports individualized, symptom-based management and emphasizes implementation of a low-FODMAP diet with dietitian involvement to improve global symptoms and quality of life.^9^ In addition, the variable effectiveness of pharmacological treatments and limited evidence for some therapeutic options may further highlight the importance of dietary approaches in IBS management.^30^These guidelines suggest that no single dietary approach is universally appropriate for all patients with IBS. The findings indicate that both low-FODMAP and gluten-free diets may improve quality of life, although their effects on symptom severity appear comparable. Therefore, dietary management should be individualized. However, the absence of subtype (IBS-C and IBS-D)-specific analyses represents an important limitation of the study.

Significant improvements in IBS-QOL scores were observed after the intervention in the gluten-free diet, low-FODMAP diet, and control groups (all *P* < .001). These findings are consistent with the previous literature. However, the improvement observed in the control group suggests that these changes may not be solely attributable to dietary interventions but may also reflect increased disease- and nutrition-related awareness, along with the beneficial effects of regular outpatient follow-up in individuals with IBS. One of the most notable findings of the study was the clinically significant reduction in the IBS Symptom Severity Score (IBS-SSS) observed in both the low-FODMAP and gluten-free diet groups. The observed decreases were not only statistically significant but also clinically meaningful. These findings are consistent with previous randomized controlled trials demonstrating that both dietary approaches can reduce symptom burden in individuals with IBS. However, the mechanisms underlying symptom improvement in the gluten-free diet group remain unclear. Current evidence suggests that these effects may not be solely attributable to gluten elimination but may also reflect broader dietary modifications and the influence of other fermentable dietary components.^31^^,^^32^ Similarly, an animal study reported that gluten-containing diets were not associated with adverse metabolic or biochemical outcomes, suggesting that the effects observed with a gluten-free diet may involve overall dietary composition rather than gluten elimination alone.^33^ The pathogenesis of IBS involves alterations in gastrointestinal motility, increased intestinal fermentation, abnormal gas accumulation, genetic and psychosocial factors, dysbiosis of the gut microbiota, and a reduction in the number and diversity of bifidobacteria in the lumen. In a study examining abdominal pain in patients with IBS following dietary interventions, both the low-FODMAP and gluten-free diet groups demonstrated significant improvements in abdominal pain scores postintervention compared with baseline.^34^ In a large-scale review including 437 patients (221 on a combined low-FODMAP and gluten-free diet, 216 on a gluten-free diet) across 4 randomized controlled trials and 4 cohort studies, the combined use of the 2 diets was associated with greater reductions in VAS bloating scores (*P* = .0010) and VAS pain scores (*P* = .005) compared with the gluten-free diet alone. Additionally, significant improvements were observed in IBS-SSS scores (*P* = .03) and IBS-QOL scores (*P* = .008).^35^ Subgroup analyses of IBS-VAS suggested that dietary interventions may exert symptom-specific effects. The low-FODMAP diet was associated with improvements in abdominal pain, bloating/gas, constipation, and psychological well-being, which may be related to reduced intestinal gas production and visceral hypersensitivity through restriction of fermentable carbohydrates. In the gluten-free diet group, improvements were observed across a broader range of symptoms, particularly diarrhea. However, these findings should be interpreted with caution, as the observed effects of the gluten-free diet may, at least in part, reflect a concurrent reduction in fermentable carbohydrate intake rather than gluten elimination alone. Taken together, although improvements were observed across dietary approaches, the underlying drivers of these effects remain difficult to disentangle. Interindividual differences in carbohydrate tolerance, intestinal permeability, and gut–brain axis sensitivity may further influence dietary response. Overall, these findings support a cautious interpretation and highlight the importance of individualized nutritional strategies rather than a uniform dietary approach in the management of IBS.

In terms of nutrient intake, a significant reduction in energy and dietary fiber intake was observed in the gluten-free diet group. Therefore, the potential reduction in dietary fiber intake associated with restrictive dietary patterns such as gluten-free diets should be considered. Although short-term symptom improvement may occur, reduced fiber intake may have implications for gut microbiota and long-term intestinal health. This highlights the importance of ensuring adequate fiber intake and careful dietary planning when implementing elimination diets.^36^In contrast, the increase in protein intake observed in the low-FODMAP diet group suggests that nutrient deficiencies in restrictive diets can be prevented with proper planning and dietary education. These findings highlight the importance of implementing elimination diets under the supervision of a dietitian.

Several studies have reported that anthropometric changes in individuals with IBS are influenced by adherence to low-FODMAP and gluten-free diets. In a study, although total daily energy intake was similar among patients with IBS, they were found to have the lowest daily FODMAP intake compared with their nonoverweight and nonobese peers. A negative correlation was observed between total FODMAP content and body fat.^37^ Higher intake in the medium- and low-FODMAP groups was associated with a lower WHR and higher fat-free mass. A long-term personalized low-FODMAP intervention has been shown to provide significant symptom improvement without leading to significant changes in body weight.^38^Therefore, the anthropometric changes observed in the present study may be attributable to the short intervention duration and transient reductions in energy intake during the elimination phase and should not be generalized to long-term body weight management. The strengths of this study include its randomized controlled design, the use of validated IBS-specific scales, and the direct comparison of 2 commonly used dietary approaches. However, the main limitations are the short duration of the intervention, the predominantly female sample, and the absence of microbiota or biomarker analyses. Future studies are recommended to include longer follow-up periods, more heterogeneous samples, and parameters that elucidate underlying biological mechanisms.

The inclusion of detailed dietary intake analysis and dietitian-led individualized nutritional counseling further enhances the clinical applicability of the findings. However, certain limitations should be acknowledged. The short intervention duration (4 weeks) may have limited the evaluation of long-term efficacy and sustainability. The predominance of female participants restricts the generalizability of the results. This study has several limitations, including the relatively small sample size, short intervention duration, lack of validated adherence assessment tools for both diets, and the absence of an intention-to-treat analysis. Although the per-protocol approach may limit generalizability, similar withdrawal rates across groups suggest a limited risk of attrition bias. Although no standardized adherence score was used, all participants received structured dietitian-led counseling and regular follow-up. The predominance of female participants in the present study (87.5%) is consistent with the female predominance commonly reported in IBS populations in the literature. In addition, the absence of microbiota and inflammatory biomarker assessments limited a more comprehensive evaluation of the biological effects of dietary interventions. Finally, the lack of symptom improvement in some participants further supports the heterogeneous response to dietary interventions in IBS. In conclusion, this study suggests that both low-FODMAP and gluten-free diets may improve gastrointestinal symptom severity and improve the quality of life in individuals with IBS in the short term. Although both dietary approaches provide clinical benefits, symptom-specific responses and potential nutrient deficiencies highlight the importance of individualized nutritional therapy. In particular, given the risk of fiber and energy inadequacies in gluten-free diets, dietary interventions should be carefully planned and monitored under the supervision of a dietitian.

## Figures and Tables

**Table 1.** Comparison of Sociodemographic Characteristics and Selected Variables Between Groups

Characteristics	Control Group (n = 21)	FODMAP Diet Group (n = 21)	Gluten-Free Diet Group (n = 21)	Test Statistic	*P*
Gender				2.005	.508
Male	3 (14.3)	1 (4.8)	4 (19.0)		
Female	18 (85.7)	20 (95.2)	17 (81.0)		
Marital status				0.917	.632
Married	10 (47.6)	7 (33.3)	9 (42.9)		
Single	11 (52.4)	14 (66.7)	12 (57.1)		
Education status				6.754	.15
High school or below	10 (47.6)	4 (19.0)	9 (42.9)		
Bachelor’s degree	8 (38.1)	16 (76.2)	10 (47.6)		
Master’s degree or below	3 (14.3)	1 (4.8)	2 (9.5)		
Income status				2.751	.596
Income less than expenses	3 (14.3)	6 (28.6)	3 (14.3)		
Income equal to expenses	9 (42.9)	10 (47.6)	10 (47.6)		
Income greater than expenses	9 (42.9)	5 (23.8)	8 (38.1)		
Employment status				3.274	.195
Yes	7 (33.3)	7 (33.3)	12 (57.1)		
No	14 (66.7)	14 (66.7)	9 (42.9)		
BMI				8.175	.226
Underweight	2 (9.5)	0	0		
Normal	10 (47.6)	16 (76.2)	14 (66.6)		
Overweight	8 (38.1)	3 (14.3)	5 (23.9)		
Obese	1 (4.8)	2 (9.5)	2 (9.5)		
Smoking status				3.675	.159
Yes	2 (9.5)	7 (33.3)	6 (28.6)		
No	19 (90.5)	14 (66.7)	15 (71.4)		
Alcohol consumption				1.128	.569
Yes	4 (22.2)	6 (28.6)	7 (33.3)		
No	17 (81.0)	15 (71.4)	14 (66.7)		
IPAQ physical activity level				8.267	.084
Inactive	7 (33.3)	9 (42.9)	14 (66.7)		
Minimally active	8 (38.1)	10 (47.6)	6 (28.6)		
Highly active	6 (28.6)	2 (9.5)	1 (4.8)		

Data are expressed as n (%). The chi-square test was used for analysis.

BMI, body mass index; IPAQ, International Physical Activity Questionnaire.

**Table 2.** Comparison of Groups in Terms of Age and Pre- and Postintervention Anthropometric Measurements, IBS-QOL, and IBS-SSS

Characteristics		Control Group (n = 21)	FODMAP Diet Group (n = 21)	Gluten-Free Diet Group (n = 21)	Effect Size (95% CI)	Test Statistics	*P*	ANCOVA Results		
								*η* ^2^	*F*	*P* ^#^
Age (years)		30.0 (22.0-47.5)	26.0 (23.0-36.5)	26.0 (24.0-34.5)		0.354	.838			
Body weight (kg)	Baseline	67.58 ± 11.44	65.09 ± 9.59	69.11 ± 12.17	0.02 (0.01-0.12)	0.700	.500	0.150	5.188	**.008**
	Week 4	67.72 ± 11.56	63.35 ± 9.22	68.15 ± 12.15	0.04 (0.01-0.15)	1.214	.304			
	Test statistics	0.962	3.044	2.202						
	P*	.348	**.006**	**.040**						
BMI (kg/m^2^)	Baseline	23.90 ± 3.49	23.49 ± 3.48	24.88 ± 3.86	0.03 (0.01-0.12)	0.820	.445	0.108	3.581	**.034**
	Week 4	23.94 ± 3.53	23.07 ± 3.17	24.57 ± 3.89	0.03 (0.01-0.13)	0.950	.393			
	Test statistics	0.762	2.780	1.969						
	P*	.455	**.012**	.063						
Waist circumference (cm)	Baseline	80.71 ± 10.45	80.17 ± 10.92	81.33 ± 10.31	0.01 (0.01-0.03)	0.064	.938	0.132	4.502	**.015**
	Week 4	80.95 ± 10.42	77.57 ± 11.17	81.00 ± 10.01	0.02 (0.01-0.12)	0.730	.486			
	Test statistics	1.558	4.986	0.297						
	P*	.135	**<.001**	.769						
Hip circumference (cm)	Baseline	100.81 ± 8.38	100.67 ± 7.38	102.62 ± 9.65	0.01 (0.01-0.08)	0.343	.711	0.026	0.798	.455
	Week 4	100.86 ± 8.33	100.24 ± 6.97	97.57 ± 21.71	0.01 (0.01-0.08)	0.326	.723			
	Test statistics	0.326	1.279	1.039						
	P*	.748	.215	.311						
Waist-to-hip ratio (cm)	Baseline	0.80 ± 0.09	0.79 ± 0.08	0.79 ± 0.08	0.01 (0.01-0.02)	0.048	.953	0.113	3.775	**.029**
	Week 4	0.80 ± 0.09	0.78 ± 0.08	0.78 ± 0.08	0.02 (0.01-0.10)	0.456	.636			
	Test statistics	1.451	2.423	1.972						
	P*	.162	**.025**	.063						
IBS-QOL	Baseline	121.76 ± 32.00^a^	104.24 ± 25.26^ab^	96.90 ± 29.55^b^	0.12 (0.01-0.26)	4.053	**.022**	0.211	7.909	**<.001**
	Week 4	130.38 ± 32.17	131.57 ± 29.38	131.10 ± 26.60	0.01 (0.01-0.01)	0.009	.991			
	Test statistics	5.035	5.677	8.116						
	P*	**<.001**	**<.001**	**<.001**						
IBS-SSS	Baseline	245 (147.5-335)	290.0 (215-420)	320.0 (257.5-372.5)	0.06 (0.02-0.27)	5.497	.064	0.071	2.252	.114
	Week 4	170 (75-250)	150.0 (62.5-300)	150.0 (50-260)	0.03 (0.03-0.11)	0.422	.810			
	Test statistics	2.176	3.598	3.823						
	P*	**.030**	**<.001**	**<.001**						

The data are expressed as mean ± SD median (first quartile–third quartile). *P* indicates the significance of the difference between groups. P* indicates the significance of the difference between time points. One-way analysis of variance, Kruskal–Wallis test, paired samples *t*-test, and Wilcoxon signed-rank test were used for the analyses. The same letters within the same row indicate similarity between groups, whereas different letters indicate significant differences between groups.

ANCOVA, analysis of covariance; BMI, body mass index; IBS-QOL, irritable bowel syndrome Quality of Life questionnaire; IBS-SSS, irritable bowel syndrome Symptom Severity Score.

# values obtained from ANCOVA after adjustment for baseline values. Bold values indicate statistically significant results (*P* < .05).

**Table 3.** Distribution of Energy, Macronutrient, and Dietary Fiber Intakes of the Groups Before and After the Intervention

Characteristics		Control Group (n = 21)	FODMAP Diet Group (n = 21)	Gluten-Free Diet Group (n = 21)	Effect Size (95% CI)	Test Statistics	*P*	ANCOVA Results		
								*η* ^2^	*F*	*P#*
Energy (kcal)	Baseline	1495.0 (1288.2-1886.5)^a^	1101.0 (996.7-1621.0)^b^	1558.3 (1253.2-1620.0)^ab^	0.11 (0.01-0.37)	8.478	**.014**	0.008	0.237	.790
	Week 4	1423.0 (1270.8-1912.5)	1199.0 (929.3-1611.2)	1351.3 (1152.8-1537.3)	0.06 (0.02-0.28)	5.699	.058			
	Test statistics	0.330	0.295	2.207						
	P*	.741	.768	**.027**						
Carbohydrates (g)	Baseline	154.0 (135.0-231.9)	112.3 (97.9-188.7)	168.6 (141.0-198.7)	0.05 (0.03-0.25)	4.819	.090	0.149	5.166	**.009**
	Week 4	172.0 (137.9-214.1)^a^	107.9 (74.6-169.8)^b^	107.9 (84.5-141.8)^b^	0.20 (0.05-0.43)	14.164	**<.001**			
	Test statistics	0.121	2.381	3.389						
	P*	.904	**.017**	**<.001**						
Protein (g)	Baseline	56.6 (47.3-86.2)^a^	47.8 (38.7-56.9)^b^	56.6 (50.9-59.9)^ab^	0.08 (0.01-0.29)	6.766	**.034**	0.067	2.121	.129
	Week 4	59.1 (48.2-77.9)	61.6 (49.1-83.4)	59.5 (47.9-83.9)	0.03 (0.03-0.10)	0.144	.931			
	Test statistics	0.608	3.007	1.755						
	P*	.543	**.003**	.079						
Fat (g)	Baseline	66.3 (54.4-83.5)^a^	48.0 (38.8-75.0)^b^	57.7 (50.7-82.7)^ab^	0.07 (0.02-0.30)	6.388	**.041**	0.023	0.710	.496
	Week 4	61.6 (50.0-92.1)	54.1 (44.3-87.2)	66.4 (55.2-86.1)	0.01 (0.01-0.17)	1.810	.405			
	Test statistics	0.504	1.512	0.261						
	P*	.614	.131	.794						
Dietary fiber (g)	Baseline	16.2 (13.6-21.0)	13.2 (9.9-19.6)	15.0 (13.0-20.6)	0.01 (0.01-0.19)	2.450	.294	0.055	1.717	.188
	Week 4	17.7 (13.3-19.3)^a^	12.0 (9.5-16.1)^b^	13.0 (10.5-16.7)^ab^	0.09 (0.01-0.032)	7.149	**.028**			
	Test statistics	0.261	1.686	1.929						
	P*	.794	.092	.054						

Data are presented as median (first quartile–third quartile). *P* values indicate significance of the difference between groups. P* values indicate significance of within-group differences across time points. Kruskal–Wallis and Wilcoxon signed-rank tests were used for the analyses. The same letters within the same row indicate similarity between groups, whereas different letters indicate significant differences between groups.

ANCOVA, analysis of covariance.

**Table 4.** Comparison of the Group Responses to IBS-Related Visual Analog Scales Before and After the Intervention

Characteristics		Control Group (n = 21)	FODMAP Diet Group (n = 21)	Gluten-Free Diet Group (n = 21)	Effect Size (95% CI)	Test Statistics	*P*	ANCOVA Results		
								*η* ^2^	*F*	*P* ^#^
Abdominal pain	Baseline	6.0 (4.0-8.0)^a^	2.0 (1.5-5.0)^b^	4.0 (0.0-5.0)^b^	0.13 (0.01-0.35)	9.733	**.008**	0.122	4.110	**.021**
	Week 4	5.0 (5.0-8.0)	9.0 (6.0-10.0)	7.0 (3.5-9.0)	0.06 (0.02-0.27)	5.583	.061			
	Test statistics	0.202	3.733	2.634						
	P*	.840	**<.001**	**.008**						
Diarrhea	Baseline	9.0 (4.5-10.0)	7.0 (4.0-10.0)	6.0 (3.5-9.0)	0.02 (0.02-0.20)	2.928	.231	0.027	0.830	.441
	Week 4	8.0 (5.5-10.0)	9.0 (7.5-10.0)	9.0 (5.0-10.0)	0.02 (0.02-0.12)	0.724	.696			
	Test statistics	0.177	1.725	2.164						
	P*	.860	.084	**.030**						
Constipation	Baseline	5.0 (2.0-8.5)^a^	2.0 (0.0-4.0)^b^	3.0 (0.0-5.5)^ab^	0.07 (0.02-0.27)	6.075	**.048**	0.185	6.696	**.002**
	Week 4	4.0 (2.0-8.0)	7.0 (3.0-9.0)	8.0 (5.0-9.0)	0.03 (0.03-0.24)	3.864	.145			
	Test statistics	0.276	3.500	3.333						
	P*	.783	**<.001**	**<.001**						
Bloating and gas	Baseline	4.0 (1.5-5.0)^a^	1.0 (0.0-3.0)^b^	1.0 (0.0-4.5)^b^	0.13 (0.01-0.36)	9.629	**.008**	0.192	7.017	**.002**
	Week 4	5.0 (1.5-5.0)^a^	7.0 (2.5-9.5)^b^	7.0 (3.0-8.5)^ab^	0.08 (0.02-0.29)	6.801	**.033**			
	Test statistics	0.001	3.852	3.352						
	P*	.999	**<.001**	**<.001**						
Nausea and vomiting	Baseline	8.0 (6.0-9.5)	8.0 (4.5-10.0)	8.0 (5.0-10.0)	0.03 (0.03-0.12)	0.327	.849	0.049	1.514	.228
	Week 4	9.0 (5.0-10.0)	10.0 (7.0-10.0)	9.0 (8.0-10.0)	0.01 (0.03-0.16)	1.643	.440			
	Test statistics	0.358	1.815	1.522						
	P*	.721	.070	.128						
Psychological status	Baseline	5.0 (2.5-7.0)	4.0 (1.0-6.5)	4.0 (2.0-7.0)	0.01 (0.01-0.16)	1.832	.400	0.166	5.876	**.005**
	Week 4	5.0 (2.5-7.0)^a^	7.0 (6.0-8.5)^b^	7.0 (4.5-9.5)^b^	0.11 (0.01-0.33)	8.337	**.015**			
	Test statistics	0.662	3.050	2.624						
	P*	.508	**.002**	**.009**						

Data are presented as median (first quartile–third quartile). *P* values indicate significance of the difference between groups. P* values indicate the significance of within-group difference across time points. Kruskal–Wallis and Wilcoxon signed-rank tests were used for the analyses. The same letters within the same row indicate similarity between groups, whereas different letters indicate significant differences between groups.

ANCOVA, analysis of covariance; IBS, irritable bowel syndrome.

## Data Availability

The data that support the findings of this study are available on request from the corresponding author.

## References

[1] Vasant DH Paine PA Black CJ British Society of Gastroenterology guidelines on the management of irritable bowel syndrome. Gut. 2021;70(7):1214 1240. (doi: 10.1136/gutjnl-2021-324598) 33903147

[2] Oka P Parr H Barberio B Global prevalence of irritable bowel syndrome according to Rome III or IV criteria: a systematic review and meta-analysis. Lancet Gastroenterol Hepatol. 2020;5(10):908 917. (doi: 10.1016/S2468-1253(20)30217-X) 32702295

[3] Can G Yılmaz B. İrritabl barsak sendromunun tanı ve tedavisinde yaklaşımlar. Guncel Gastroenterol. 2015;19(3):171 178.

[4] Yılmaz B Akbulut G. The evaluation of the nutritional status in patients with irritable bowel syndrome. Clin Exp Health Sci. 2021;11(1):119 126. (doi: 10.33808/clinexphealthsci.646176)

[5] Yeşil E Başerdem N. FODMAP diet treatment for irritable bowel syndrome. Turk J Curr Gastroenterol. 2023;25:187 191.

[6] Marano G Traversi G Pola R Irritable bowel syndrome: a hallmark of psychological distress in women? Life. 2025;15(2):277. (doi: 10.3390/life15020277) 40003686 PMC11856493

[7] Black CJ Ford AC. An evidence-based update on the diagnosis and management of irritable bowel syndrome. Expert Rev Gastroenterol Hepatol. 2025;19(3):227 242. (doi: 10.1080/17474124.2025.2455586) 39835671

[8] Ghega H Ali Z Sohail S Efficacy of dietary interventions in managing irritable bowel syndrome: a systematic review of clinical outcomes and gut microbiota modulation. Cureus. 2025;17(8):e90889. (doi: 10.7759/cureus.90889) PMC12456667 40995291

[9] Choi Y Youn YH Kang SJ 2025 Seoul consensus on clinical practice guidelines for irritable bowel syndrome. J Neurogastroenterol Motil. 2025;31(2):133 169. (doi: 10.5056/jnm25007) 40205893 PMC11986658

[10] Cuffe MS Staudacher HM Aziz I Efficacy of dietary interventions in irritable bowel syndrome: a systematic review and network meta-analysis. Lancet Gastroenterol Hepatol. 2025;10(6):520 536. (doi: 10.1016/S2468-1253(25)00054-8) 40258374

[11] Chey WD Hashash JG Manning L AGA clinical practice update on the role of diet in irritable bowel syndrome. Gastroenterology. 2022;162(1):219 227. 10.1053/j.gastro.2021.12.248 35337654

[12] Van den Houte K Colomier E Routhiaux K Efficacy and findings of a blinded randomized reintroduction phase for the low FODMAP diet in irritable bowel syndrome. Gastroenterology. 2024;167(2):333 342. (doi: 10.1053/j.gastro.2024.02.008) 38401741

[13] Siragusa N Baldassari G Ferrario L The ten dietary commandments for patients with irritable bowel syndrome: a narrative review with pragmatic indications. Nutrients. 2025;17(15):2496. (doi: 10.3390/nu17152496) PMC12348238 40806081

[14] Colgan A Digby K Apekey T A dietitian-led low-FODMAP diet webinar: a pre-post study evaluating its impact on symptoms of irritable bowel syndrome. J Hum Nutr Diet. 2024;37(2):396 407. (doi: 10.1111/jhn.13262) 37905715

[15] Paduano D Cingolani A Tanda E Effect of three diets (low-FODMAP, gluten-free and balanced) on irritable bowel syndrome symptoms and health-related quality of life. Nutrients. 2019;11(7):1566. (doi: 10.3390/nu11071566) PMC6683324 31336747

[16] Rakıcıoğlu N Tek N Ayaz A Yemek ve Besin Fotoğraf Kataloğu: Ölçü ve Miktarlar. Ankara, Turkey: Ata Ofset; 2010.

[17] Francis CY Morris J Whorwell PJ. The irritable bowel severity scoring system: a simple method of monitoring irritable bowel syndrome and its progress. Aliment Pharmacol Ther. 1997;11(2):395 402. (doi: 10.1046/j.1365-2036.1997.142318000.x) 9146781

[18] Patrick DL Drossman DA Frederick IO Quality of life in persons with irritable bowel syndrome: development and validation of a new measure. Dig Dis Sci. 1998;43(2):400 411. (doi: 10.1023/A:1018831127942) 9512138

[19] Uran BNO Karadakovan A Vardar R Bor S. Psychometric Properties of the Irritable Bowel Syndrome Quality of Life Scale in Turkey. J Hepatol Gastroint Dis. 2016;2(3):137.

[20] Bengtsson M Persson J Sjölund K Further validation of the visual analogue scale for irritable bowel syndrome after use in clinical practice. Gastroenterol Nurs. 2013;36(3):188 198. (doi: 10.1097/SGA.0b013e3182945881) 23732784

[21] Bellini M Tonarelli S Mumolo MG Low fermentable oligo- di- and mono-saccharides and polyols (FODMAPs) or gluten free diet: what is best for irritable bowel syndrome? Nutrients. 2020;12(11):3368. (doi: 10.3390/nu12113368) PMC7692077 33139629

[22] Akbulut G Tıbbi Beslenme Tedavisinde Güncel Uygulamalar-I: Besin Alerjisi, Besin İntoleransı Durumlarında Beslenme ve Test Diyetleri. Ankara, Turkey: Ankara Nobel Tıp Kitabevi; 2016.

[23] Lacy BE Pimentel M Brenner DM ACG Clinical Guideline: management of irritable bowel syndrome. Am J Gastroenterol. 2021;116(1):17 44. (doi: 10.14309/ajg.0000000000001036) 33315591

[24] Sağlam M Arıkan H Savcı S International Physical Activity Questionnaire: reliability and validity of the Turkish version. Percept Mot Skills. 2010;111(1):278 284. (doi: 10.2466/06.08.PMS.111.4.278-284) 21058606

[25] Turan B Bengi G Cehreli R Clinical effectiveness of adding probiotics to a low FODMAP diet: randomized double-blind placebo-controlled study. World J Clin Cases. 2021;9(25):7417 7432. (doi: 10.12998/wjcc.v9.i25.7417) 34616808 PMC8464468

[26] Dimidi E Whelan K. Food supplements and diet as treatment options in irritable bowel syndrome. Neurogastroenterology Motil. 2020;32(8):e13951. (doi: 10.1111/nmo.13951) 32697018

[27] Liu J Chey WD Haller E Low-FODMAP diet for irritable bowel syndrome: what we know and what we have yet to learn. Annu Rev Med. 2020;71(1):303 314. (doi: 10.1146/annurev-med-050218-013625) 31986083

[28] Ustaoğlu T Acar Tek N Yıldırım AE. İrritabl bağırsak sendromunda (İBS) FODMAP diyetinin İBS semptomları, beslenme durumu ve yaşam kalitesi üzerine etkilerinin değerlendirilmesi. Beslenme Diyet Derg. 2020;48(1):47 56.

[29] Eswaran S Chey WD Jackson K A diet low in fermentable oligo-, di-, and monosaccharides and polyols improves quality of life and reduces activity impairment in patients with irritable bowel syndrome and diarrhea. Clin Gastroenterol Hepatol. 2017;15(12):1890 1899.e3. (doi: 10.1016/j.cgh.2017.06.044) 28668539

[30] Bor S Özen H Kalkan İH Expert opinion on the efficacy and safety of antispasmodics with a focus on irritable bowel syndrome. Turk J Gastroenterol. 2025;36(suppl 2):S1 S38. (doi: 10.5152/tjg.2025.101125) PMC13007261 41866875

[31] Asghar W Khalid N. Low FODMAP diets—boon or bane for individuals with GI disorders. Nutr Health. 2024;30(4):639 640. (doi: 10.1177/02601060241297749) 39529351

[32] Herfindal AM Nilsen M Aspholm TE Effects of fructan and gluten on gut microbiota in individuals with self-reported non-celiac gluten/wheat sensitivity—a randomised controlled crossover trial. BMC Med. 2024;22:251. 10.1186/s12916-024-03562-1 PMC11373345 39227818

[33] Bektaş A Ulusoy M Özsarı L The effects of gluten on weight gain, hematological, biochemical, and various endocrinological parameters. Turk J Gastroenterol. 2024;35(3):178 185. (doi: 10.5152/tjg.2024.23210) 39128121 PMC11059451

[34] Yoo HY Park B Joo J Validation of the Korean version of visual analogue scale for irritable bowel syndrome questionnaire for assessment of defecation pattern changes. Ann Surg Treat Res. 2018;94(5):254 261. (doi: 10.4174/astr.2018.94.5.254) 29732357 PMC5931936

[35] Zhang J Yu P Xu Y Efficacy and safety of a low-FODMAP diet in combination with a gluten-free diet for adult irritable bowel syndrome: a systematic review and meta-analysis. Dig Dis Sci. 2024;69(11):4124 4132. (doi: 10.1007/s10620-024-08671-8) 39407084

[36] Silva H Porter J Barrett J Dietary intake, symptom control and quality of life after dietitian-delivered education on a FODMAP diet for irritable bowel syndrome: a 7-year follow up. Neurogastroenterology Motil. 2025;37(12). (doi: 10.1111/nmo.70116) PMC12623287 40589416

[37] Chu N He J Ling J Higher habitual FODMAP intake is associated with lower body mass index, lower insulin resistance and higher short-chain fatty acid-producing microbiota in people with prediabetes. bioRxiv. (doi: 10.1101/2022.10.26.513956)

[38] Staudacher HM Rossi M Kaminski T Long-term personalized low FODMAP diet improves symptoms and maintains luminal bifidobacteria abundance in irritable bowel syndrome. Neurogastroenterology Motil. 2022;34(4):e14241. (doi: 10.1111/nmo.14241) 34431172

